# The Quantum Zeno Capacity and Dynamic Evolution Mode of a Quantum System

**DOI:** 10.3390/e26121080

**Published:** 2024-12-11

**Authors:** Zhenbo Ni, Yonggang Peng, Yujun Zheng

**Affiliations:** School of Physics, Shandong University, Jinan 250100, China; realmilan@126.com (Z.N.); ypeng@sdu.edu.cn (Y.P.)

**Keywords:** quantum Zeno effect, quantum engineering, quantum Zeno factor

## Abstract

The quantum Zeno effect (QZE) is widely employed in quantum engineering due to the issue of frequent measurements freezing a quantum system. In this study, the quantum Zeno factor is introduced to characterize the quantum Zeno capacity of a quantum system. The quantum Zeno factor reveals that the quantum Zeno effect is dependent on the evolution mode of quantum states, which is semi-irrelevant to conventional energy uncertainty and extends the QZE domain. The Zeno factor provides a new consideration to qualify the (anti-)Zeno capacity of a quantum system for its applications: a large quantum Zeno factor value indicates that a quantum system is of a QZE quality. The numerical results of the quantum Zeno capacity are shown using two typical examples: tailing the dynamic evolution modes using the quantum Zeno factor in a three-level system, and quantifying the message exchange between qubits in a coupled qubit system using a quantum Zeno factor.

## 1. Introduction

The Zeno phenomenon in quantum physics was introduced by von Neumann in his study of short time evolution [1]. Misra and Sudarshan discovered that if the unstable system was frequently monitored, the system decayed slower than the anticipated exponential decay law at a sufficiently short length of time, and the back action of the measurement led back to the initial state, which shed light on the Zeno paradox in quantum theory [2,3,4,5]. Since then, the concept of the quantum Zeno effect in quantum systems has come to light, and there has been considerable interest in the phenomena of frequent measurements freezing states. In contrast to the frozen evolution state, measurements have been recently developed to also enhance the decay rate under more general conditions denoted by the quantum anti-Zeno effect (QAZE) [6,7,8]. Cook [9] put forward an experimental proposal for observing the quantum Zeno effect in a three-level atomic transition, and Itano et al. [10] experimentally demonstrated that frequent measurements inhibit quantum jumps. Since then, experimental and theoretical investigations of the quantum Zeno effect have extended to various scenarios, such as unstable state tunneling in optical lattices [11,12], measurement-induced phase transition in quantum many-body systems [13,14,15], and heating up or cooling down processes in a qubit-bath system [16,17].

Interestingly, the quantum Zeno effect, a powerful tool in quantum engineering, and quantum system measurements are widely studied [13,14,15,18,19,20,21,22,23]. The measurements are not limited to the initial state but extend to a projection onto a multidimensional subspace where the system is manipulated to evolve as expected, demonstrating the quantum Zeno dynamics (QZD) [24,25,26,27,28,29,30]. Furthermore, the measurement is not a compulsory ingredient for achieving the quantum Zeno effect as the theoretical predictions and experimental realizations [24,25,31,32,33]. Except for frequent projective measurements, bang–bang decoupling, strong continuous coupling, and strong damping are also regarded as the manifestations of the quantum Zeno effect [25,31,32]. The quantum Zeno effect or quantum Zeno dynamics have attracted considerable attention to control the quantum system due to the deceleration or acceleration features, for instance, in quantum computation [34,35,36], quantum coherence and entanglement [8,28,37,38,39,40,41,42], thermodynamic control [16,43,44], and quantum walks [27,45,46,47]. In addition, the dynamic projective measurement with a closed loop in projective Hilbert space, which leads to geometric phase accumulation, is theoretically and experimentally investigated by applying the dynamic quantum Zeno effect [48,49].

It is well known that a quantum system can dynamically evolve in different ways in Hilbert space by employing the gauge transformation freedom of a quantum system; for example, the quantum system could be in phase or parallel evolution. Here, we define them as the evolution modes of a quantum system. The multifarious dynamic evolution modes of the quantum system originate from the gauge transformation freedom of the quantum states in Hilbert space [50,51]. Many efforts have been devoted to investigating the physical mechanism and the potential applications of the quantum (anti-)Zeno effect in the quantum engineering community [8,31,34,35,37,38,52]. However, studies on the physical mechanism of the (anti-)Zeno effect and its usability in quantum engineering have not yet focused on the intrinsic relationship between the dynamic evolution modes and the (anti-)Zeno effects. Since the gauge transformation freedom of the quantum states in Hilbert space is related to differential geometry, geometric ideas have been shown to enrich our understanding of quantum theory and reveal different considerations in the quantum engineering community.

In this study, we geometrically present the generalized framework of the quantum Zeno effect, which is dependent on the nature of the dynamic evolution modes of a quantum system within a short time period. The QZE domain is extended or is deemed semi-irrelevant to conventional energy uncertainty. We introduce the quantum Zeno factor, defined as the inverse of the quantum speed limits, to characterize the quantum Zeno capacity of a quantum system. We consider the quantum Zeno effects as the representation of dynamic evolution behaviors in a short time period and examine its dynamic evolution behaviors by employing the geometric properties of quantum mechanics. We establish the relation between the quantum Zeno effect and factor. Our investigation presents the high-efficient usability of the quantum Zeno effect via choosing different dynamic evolution modes in the quantum engineering community and different ways to control the quantum Zeno effect.

This study is organized as follows: in Section 2.1, we present the quantum Zeno effect related to the Hilbert space distance; to characterize the quantum Zeno capacity of a quantum system, the quantum Zeno factor is introduced in Section 2.2; in Section 2.3, we discuss the method of controlling the quantum Zeno effect by tailoring the evolution curves corresponding to different evolution modes or quantum speed limit bounds; two typical examples of the quantum Zeno effect are studied in Section 3; our concluding remarks are outlined in Section 4.

## 2. Theoretical Framework

### 2.1. Geometric Framework of Generalized Quantum Zeno Effect

The quantum Zeno effect describes the dynamic evolution of a quantum system induced by frequent measurements, leading back to the initial state. We consider a quantum system (with the Hermitian Hamiltonian H(t)) with a dynamic evolution described in the Hilbert space H. The initial state of the system is prepared at $|\psi_{i}\rangle$, and its dynamic evolution is governed by the Schrödinger equation as follows:

(1) $$i\hbar \frac{d}{dt}|\psi \left(t\right)\rangle =H\left(t\right)|\psi \left(t\right)\rangle .$$

Thus, the state at time *t* is $\left|\psi \left(t\right)\rangle \equiv \right|\psi_{t}\rangle =U\left(t\right)|\psi_{i}\rangle$, and $U\left(t\right)=Te^{-\frac{i}{\hbar }\int_{0}^{t}H\left(t^{\prime }\right)dt^{\prime }}$ is the dynamic evolution operator. Then, the survival probability is defined as follows:

(2) $$S\left(t\right)=|\langle \psi_{i}|\psi_{t}\rangle {|}^{2}.$$

As theoretically predicted and experimentally realized, the quantum Zeno effect can be equivalently achieved via different theoretical formalisms, such as frequent projective measurements, frequent unitary kicks, strong continuous coupling, and strong damping [25,31,32]. In this study, we suppose the system is detected *n* times with the intervals τ=t/n using projective measurements $D=|\psi_{i}\rangle \langle \psi_{i}|$ (the intervals τ are in Dyson series because of the time-ordering operator). Therefore, the survival probability of the system, after taking *n* projective measurements in the time period of [0,t], becomes

(3) $$\begin{array}{cc} S(t) & =|\langle \psi_{i}\left|UDU\cdots UDU\right|\psi_{i}{\rangle |}^{2}\\ \; & =|\langle \psi_{i}|\psi_{\tau }\rangle {|}^{2n}, \end{array}$$

where $|\psi_{\tau }\rangle =U\left(\tau \right)|\psi_{i}\rangle$ is the quantum state after evolving τ. Consequently, the quantum Zeno effect can be described by the survival probability, which is in close proximity to the overlap between the two states.

By employing the geometrical formulation of quantum mechanics, the distance between two neighboring quantum states |ψ(t)〉 and |ψ(t+τ)〉 (for small τ) can be defined via their inner product using the Fubini–Study metric, as follows [53,54,55,56]:

(4) $$ds^{2}=1-{\left|\langle \psi \left(t\right)|\psi \left(t+\tau \right)\rangle \right|}^{2}.$$

From Equations (3) and (4), we can obtain the survival probability S(t) between two neighboring states, with small τ, in relation to their distance:

(5) $$\begin{array}{cc} S(t) & ={\left({\left|\langle \psi \left(t\right)|\psi \left(t+\tau \right)\rangle \right|}^{2}\right)}^{n}\\ \; & ={\left(1-ds^{2}\right)}^{n}. \end{array}$$

Moreover, the distance ds between the neighboring states |ψ(t)〉 and |ψ(t+τ)〉 can also be expressed as follows [53,57]:

(6) $$ds=v_{QSL}\tau ,$$

where $v_{QSL}$ is the quantum speed limits (QSLs) of a quantum system from the quantum state |ψ(t)〉 to |ψ(t+τ)〉. The survival probability, after including Equations (5) and (6), turns into the following form (for small τ):

(7) $$S\left(t\right)={\left(1-v_{QSL}^{2}\tau^{2}\right)}^{n}.$$

Equation (7) demonstrates that the quantum Zeno effect would be affected by two aspects: the short time period (or the projective measurement times) and the dynamic evolution modes of the quantum system denoted by their evolution speed limits $v_{QSL}$. Alternatively, the quantum Zeno effect can be determined using the different dynamic evolution modes under a constant measurement interval time. The dynamic evolution modes can be presented through their corresponding quantum speed limit bounds [58]. Based on the gauge transformation freedom of the quantum states, the quantum state can evolve along a specific curve to realize the quantum speed limit bound. The quantum speed limits of the quantum system have been investigated [59], and some typical quantum speed limit bounds have been developed, such as the bounds related to energy uncertainty, average energy, and parallel transport developed by Mandelstam and Tamn [60], Margolus and Levitin [61], and Sun and Zheng [57], respectively. Therefore, we realize different quantum Zeno effects through three evolution curves or modes. Moreover, phase accumulation arises from the evolution modes or curves. For instance, only geometric phases accumulate in the parallel transport mode, which is usually considered a robust operation.

### 2.2. Quantum Zeno Factor

Here, we introduce quantum Zeno factor $Z_{f}$ to describe the quality of a quantum system reaching the quantum Zeno effect. The survival probability of Equation (7) can be rewritten as follows:

(8) $$\begin{array}{c} S\left(t\right)={\left(1-\frac{\tau^{2}}{Z_{f}^{2}}\right)}^{n}, \end{array}$$

where

(9) $$Z_{f}=1/v_{QSL}.$$

Furthermore, Equation (8) can, the quantum Zeno effect as the small time period effect of a quantum system, be approximately expressed as follows:

(10) $$\begin{array}{c} S\left(t\right)≃1-\frac{n}{Z_{f}^{2}}\tau^{2}≃e^{-\left(\frac{n}{Z_{f}^{2}}\right)\tau^{2}}. \end{array}$$

Equation (9) is defined as the quantum Zeno factor, which represents the capability of a quantum system in achieving the quantum Zeno effect: The higher the $Z_{f}$ value for a quantum system, the lower the amount of projective measurements. Alternatively, in the case of the same measurement times *n*, the big value of the quantum Zeno factor can obtain the “good” Zeno. It provides us with a factor to characterize the Zeno ability of a quantum system. In addition, the quantum Zeno factor $Z_{f}$, defined as the inverse of the quantum speed limit, shows that the quantum Zeno effect of a quantum system is related to its dynamic evolution modes. Correspondingly, the quantum Zeno time [24,62,63] can be written as $\tau_{QZ}=Z_{f}/\sqrt{n}$ using the quantum Zeno factor $Z_{f}$.

For a fixed evolution time *T*, if a quantum system could reach its Zeno effect, we should take enough measurement times.The minimum measurement times can be estimated by supposing $\tau <\tau_{QZ}$. For a quantum system reaching its Zeno effect, using Equation (8), we obtain the Zeno measurement times $n_{QZ}=\frac{T^{2}}{Z_{f}^{2}}$. This represents that if a quantum system is of a high quantum Zeno factor, it can reach quantum Zeno using less measurement times.

### 2.3. Controlling the Quantum Zeno Effect

We establish the relation between the quantum Zeno effect and the factor. The quantum Zeno factor of Equation (9), as the intrinsic parameter of a quantum system reaching the quantum Zeno effect, is defined by the QSL, revealing a potential method to control the quantum (anti-)Zeno effect with the QSL. Based on previous investigations for the QSL, there are different ways to control the quantum (anti-)Zeno effect in its usability for quantum engineering [64,65]. For example, a multiqubit system under Markovian dephasing channels speeds up the quantum evolution [65], the non-Markovianity can speed up quantum evolution [66,67], the quantum entanglement enhances the evolution speed [68,69], and the nonequilibrium feature of the environment can speed up the quantum evolution in both Markovian and non-Markovian dynamics regions [70].

In addition, this provides us ways to manipulate the quantum coherence, the entanglement, etc., of the quantum system by employing the quantum (anti-)Zeno effect.

## 3. Numerical Results

In this section, our formalism of the quantum Zeno effect will be presented using two typical examples. The following three dynamic evolution modes are considered for the numerical results: the conventional dynamic evolution of the quantum system accumulating both dynamic and geometric phases corresponding to the Mandelstan–Tamn QSL [60], i.e., the MT mode; the dynamic evolution without the accumulation of geometric phases corresponding to the Margolus–Levitin QSL [61], i.e., the ML mode; and the parallel transport corresponding to QSL quantified using the changing rate of the geometric phase developed by Sun and Zheng [57], i.e., the SZ mode.

### 3.1. Three-Level System

To demonstrate the quantum Zeno effect under Equation (8), we consider a three-level system, a typical model used to experimentally observe the quantum Zeno effect [10,58,71].

The system consists of a family of states as follows:

(11) $$|\psi \left(0\right)\rangle =c_{0}|E_{0}\rangle +c_{1}|E_{1}\rangle +c_{2}|E_{2}\rangle ,$$

where $|E_{j}\rangle$(j=0,1,2), with corresponding eigenvalues $E_{j}$, are the eigenstates. $c_{j}$ are the initial partitions of the eigenstates in the initial state |ψ(0)〉. Following the suggestions of Ref. [71], the eigenstates are supposed as nondegenerates and $E_{0}=0$ for the ground state $|E_{0}\rangle$.

In our numerical calculations, we scale the dynamic evolution time by the time, $\tau_{⊥}$ required for a quantum state evolving to its orthogonal state. In particular, we suppose the system evolves to its orthogonal state $|\psi (\tau_{⊥})\rangle$ from the initial state |ψ(0)〉 at time $\tau_{⊥}$, i.e., $\langle \psi \left(0\right)|\psi \left(\tau_{⊥}\right)\rangle =0$. The dynamic evolution of the system from the initial state of Equation (11) is governed using the Schrödinger equation of Equation (1). Correspondingly, the state at time t(0<t<1) can be written as follows (*ℏ* is set to unity for simplicity):

(12) $$\begin{array}{c} |\psi \left(t\right)\rangle =c_{0}|E_{0}\rangle +c_{1}e^{-iE_{1}t}|E_{1}\rangle +c_{2}e^{-iE_{2}t}|E_{2}\rangle . \end{array}$$

For this example, we consider the MT and ML evolution modes.

(1) The MT dynamic evolution mode—$Z_{f}^{MT}>Z_{f}^{ML}$.

Following Ref. [71], this dynamic evolution mode can be achieved by setting the initial distributions as follows (assumption δ≪1): $p_{0}=\frac{\delta }{2}$, $p_{1}=\frac{1}{2}-\frac{\delta }{4}\left(1+\cos x_{1}\right)$, and $p_{2}=\frac{1}{2}-\frac{\delta }{4}\left(1-\cos x_{1}\right)$, with $p_{j}={\left|c_{j}\right|}^{2};\;\sum_{j}p_{j}=1$; and $x_{j}=E_{j}\tau_{⊥}$ with the indexes j={0,1,2}.

The quantum Zeno factor of the dynamic evolution modes ML $Z_{f}^{ML}$ and MT $Z_{f}^{MT}$ can be written as follows (see Appendix A):

(13) $$\begin{array}{cc} Z_{f}^{ML} & =\frac{4}{\left(E_{1}+E_{2}\right)\left(2-\delta \right)+\left(E_{2}-E_{1}\right)\delta \cos x_{1}},\\ Z_{f}^{MT} & =\frac{2}{\sqrt{{\left(E_{1}-E_{2}\right)}^{2}+2E_{1}E_{2}\delta }}. \end{array}$$

(2) The ML dynamic evolution mode—$Z_{f}^{ML}>Z_{f}^{MT}$.

In this situation, the populations are supposed as [71] $p_{0}=\frac{1}{2}$, $p_{1}=\frac{1}{2}\left(1-\frac{\beta }{k^{2}}\right)$, $p_{2}=\frac{\beta }{2k^{2}}$. Following Ref. [71], it can be guaranteed that the initial state and the state at time $\tau_{⊥}$ are orthogonal under the condition 1/k≪1. Also, the two excited state energies have the relation of $E_{2}=\left(2k+1\right)E_{1}$, $k\in N^{*}$.

The quantum Zeno factor of the dynamic evolution modes ML $Z_{f}^{ML}$ and MT $Z_{f}^{MT}$ can be expressed as follows (see Appendix A):

(14) $$\begin{array}{cc} Z_{f}^{ML} & =\frac{2k}{\left(k+2\beta \right)E_{1}},\\ Z_{f}^{MT} & =\frac{2\sqrt{k}}{E_{1}\sqrt{k+4\beta +8k\beta }}. \end{array}$$

(3) Numerical Results. The quantum Zeno effect is described using the survival probability of Equation (8). For the measurement times $n\gg n_{QZ}$, the measurement time interval τ is infinitesimal, and the quantum Zeno effect can be obtained by the geometrical survival probability Equation (8). In order to fully demonstrate the quantum Zeno effect of different evolution modes, the quantum Zeno effect, with a small amount of measurement during the evolution, can be calculated by the definition of the survival probability. For a constant τ, the survival probability is determined by the quantum Zeno factor $Z_{f}$. In this three-level system, we consider two typical evolution modes: the dynamic evolution modes MT and ML corresponding to the quantum Zeno factors $Z_{f}^{MT}$ and $Z_{f}^{ML}$, respectively.

**Figure 1:**
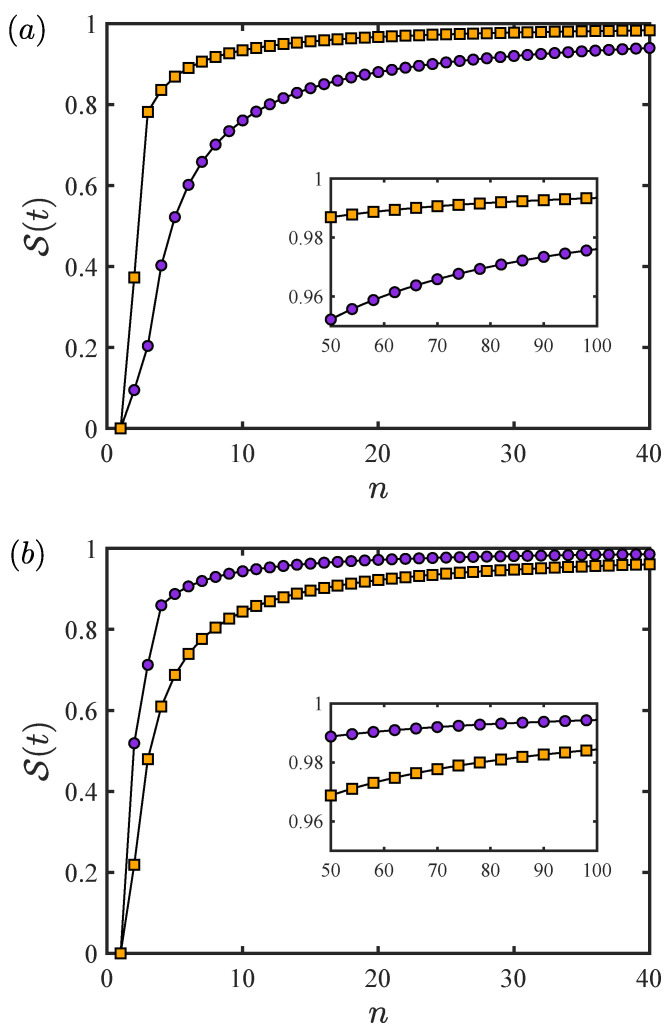
The survival probability between the initial and final state as a function of the measurement times *n*. The measurement interval is determined by τ=t/n. The upper bound speeds are depicted by orange squares and purple circles, corresponding to the energy uncertainty ΔE and the energy average 〈E〉, respectively. (**a**) The parameters used are $E_{1}=1,\;E_{2}=5,\;\delta =0.1$ and $E_{1}=1,\;E_{2}=5,\;\delta =0.02$, respectively. The quantum Zeno factors are $Z_{f}^{MT}=0.49$ and $Z_{f}^{ML}=0.34$, respectively. (**b**) The parameters used are $E_{1}=0.5,\;\beta =2,\;k=8$ and $E_{1}=1,\;\beta =1,\;k=4$, respectively. The quantum Zeno factors are $Z_{f}^{MT}=0.94$ and $Z_{f}^{ML}=1.33$, respectively.

The survival probabilities of Equation (8) can be obtained using Equations (13) and (14). The numerical results of the MT and ML dynamic evolution modes are shown in Figure 1. As graphically illustrated in Figure 1, the final state arrives at the orthogonal state when we perform a measurement at the end of the evolution time. In the case of processes involving multiple measurements, the final state always fails to arrive at the orthogonal state. However, this does not mean that all these processes are the quantum Zeno effect. It can only be considered to achieve the quantum Zeno effect with $n>n_{QZ}$, which is named as the Zeno realm. Figure 1a is interpreted as the case of MT dynamic evolution mode, i.e., the quantum Zeno factor of the MT dynamic evolution mode is bigger than that of the ML dynamic evolution mode. Apparently, the MT dynamic evolution mode can reach Zeno “earlier” than the ML dynamic evolution mode since $Z_{f}^{MT}>Z_{f}^{ML}$. Conversely, in Figure 1b, we provide a different case: $Z_{f}^{ML}>Z_{f}^{MT}$, i.e., the ML dynamic evolution mode can obtain its Zeno “earlier” than that of the MT dynamic evolution mode.

Therefore, we can exploit or avoid the quantum Zeno effect by tailoring the dynamic evolution mode using the quantum Zeno factor $Z_{f}$.

### 3.2. Coupled Qubit System

The parallel transporting of the quantum states plays a vital role in the community of quantum engineering, such as quantum computing, quantum metrology, etc. [72,73]. Here, we consider the quantum Zeno effect, employing the parallel transporting mode and the other two studied modes, in coupled qubits by frequent measurements. The coupled qubits in superconducting qubits are promising candidates for realizing a quantum computer. Meanwhile, the two-qubit gates as the universal gate with the single-qubit gate can implement any unitary transformation in a quantum computer [74,75,76,77,78].

The Hamiltonian of a coupled qubits system that is coupled to a dissipative environment is given as follows [77,78,79]:

(15) $$H=-\frac{1}{2}\left(\begin{array}{cccc} \epsilon -s+\kappa & \eta & 0 & -\Delta \eta \\ \eta & -\kappa & \eta & \Delta \epsilon -\Delta s\\ 0 & \eta & \kappa +s-\epsilon & \Delta \eta \\ -\Delta \eta & \Delta \epsilon -\Delta s & \Delta \eta & -\kappa \end{array}\right),$$

with $\epsilon =\epsilon_{1}+\epsilon_{2}$, $\Delta \epsilon =\epsilon_{1}-\epsilon_{2}$, $\eta =\left(\gamma_{1}+\gamma_{2}\right)/\sqrt{2}$, and $\Delta \eta =\left(\gamma_{1}-\gamma_{2}\right)/\sqrt{2}$, where *ϵ_ı_* (*ı* =1,2) are the energy bias, and γ_*ı*_ is the transmission amplitude through the barrier. κ is the strength of the inter-qubit coupling, and $s=X_{1}+X_{2}$ and $\Delta s=X_{1}-X_{2}$, where X_*ı*_ is the coordinate of the bath of harmonic oscillators. It is usually supposed that the system is composed of two equal qubits, namely, Δη=Δϵ=Δs=0 [77,78,79].

For the system of two equal qubits, we employ a superposition of the eigenstates as the initial state of the following:

(16) $$\begin{array}{c} |\psi \left(0\right)\rangle =c_{1}|\xi_{1}\rangle +c_{2}|\xi_{2}\rangle +c_{3}|\xi_{3}\rangle +c_{4}|\xi_{4}\rangle , \end{array}$$

where $|\xi_{j}\rangle$ (j=1,⋯,4) are the eigenstates with the eigenvalues $\xi_{j}$.

The state at time *t* is

(17) $$\begin{array}{cc} |\psi (t)\rangle & =\sum_{j=1}^{4}c_{j}e^{-i\xi_{j}t}|\xi_{j}\rangle . \end{array}$$

Consequently, the quantum Zeno factor of the ML and MT dynamic evolution modes can be written as follows:

(18) $$\begin{array}{cc} Z_{f}^{ML} & =\frac{1}{\sum_{j=1}^{4}p_{j}\xi_{j}},\\ Z_{f}^{MT} & =\frac{1}{\sqrt{\sum_{j=1}^{4}p_{j}\xi_{j}^{2}-{\left(\sum_{j=1}^{4}p_{j}\xi_{j}\right)}^{2}}}, \end{array}$$

where the weight coefficient satisfied $p_{j}={\left|c_{j}\right|}^{2}$.

We include the parallel transporting mode in the coupled-qubit system. The quantum Zeno factor of the parallel transporting of the coupled-qubit system can be obtained using the QSL bound of the parallel transporting developed by Sun and Zheng in Ref. [57]:

(19) $$Z_{f}^{SZ}=1/\left|{\dot{\varphi }}_{geo}\right|,$$

and the changing rate of the geometric phase can be calculated using [80]

(20) $$\begin{array}{c} \varphi_{geo}=\sum_{j=1}^{4}p_{j}\xi_{j}t+\arctan\frac{\sum_{j=1}^{4}p_{j}\sin\xi_{j}t}{\sum_{j=1}^{4}p_{j}\cos\xi_{j}t}. \end{array}$$

**Figure 2:**
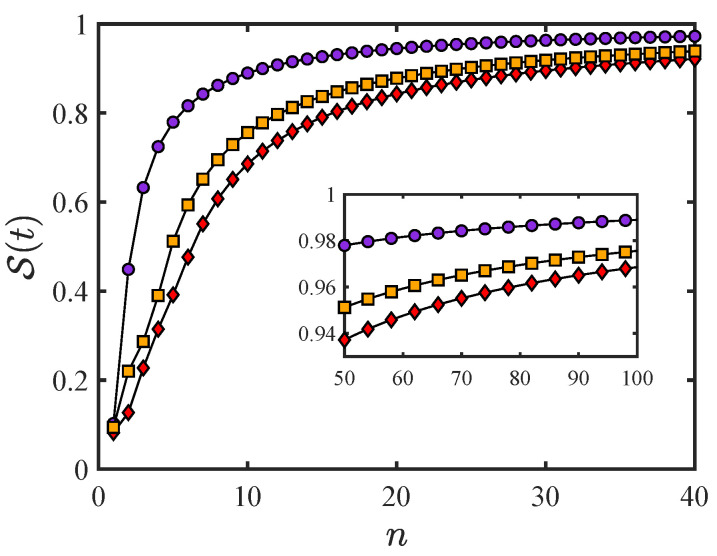
The survival probability of the system containing two ferromagnetic coupling qubits. The orange squares are given by evolving along a conventional curve, $\epsilon =\eta =1,\;\kappa =1.2,\;c_{1}=0.5,\;$$c_{2}=0.13,\;c_{3}=0.52,\;c_{4}=0.68,\;Z_{f}^{MT}=1.64$. The path only including the dynamic phase is the purple one, $\epsilon =1.5,\;\eta =1,\;\kappa =0.5,\;c_{1}=0.5,\;c_{2}=-0.13,\;c_{3}=0.5,\;c_{4}=0.7,\;Z_{f}^{ML}=2.04$. Obviously, the remaining curve corresponds to the geometric phase, $\epsilon =0.5,\;\eta =0.3,\;\kappa =1.1,\;c_{1}=0.5,\;$$c_{2}=0.35,\;c_{3}=0.55,\;c_{4}=0.57,\;Z_{f}^{SZ}=1.18$.

To show the numerical results of this case, the initial state takes the maximum coherence state $|\psi \left(0\right)\rangle =\frac{1}{2}{\left(1,1,1,1\right)}^{T}$. The other parameters are shown in the caption of Figure 2. The survival probability can be obtained with three quantum Zeno factors of the quantum system (see Appendix A).

The numerical results of the quantum Zeno effect of the two coupled qubits are shown in Figure 2. The results of the MT and ML dynamic evolution modes and the SZ mode (for parallel transporting) are depicted by orange squares, purple circles, and red diamonds, respectively. As shown in the inset, all three modes have a survival probability close to the unit, which indicates that a sufficiently high measurement frequency can completely freeze the quantum state. That is, the infinitesimal measurement time interval τ in Equation (8) can “wipe out” everything of the dynamic evolution. However, in the intermediate τ, the differences in the dynamic evolution modes are sufficient to affect the measurement-induced deceleration of the quantum system evolution. As shown in Figure 2 using red diamonds, the SZ mode for the system has the smallest quantum Zeno factor, which means that the SZ mode for the system effectively resists the measurement-induced slowdown effect. Alternatively, the SZ mode is insufficient to result in the quantum Zeno effect. This demonstrates that frequent projective measurements between the quantum qubits change between the quantum hard disk and a computational device, and simultaneously sustain efficient information regarding reading and writing interactions in the computation process. In particular, the SZ mode could allow us to effectively avoid the quantum Zeno effect to obtain more exchanges in information within the system.

## 4. Conclusions

The quantum Zeno effect, as the typical dynamics of a quantum state within a short period of time, is geometrically investigated. We present that the quantum Zeno effect is dependent on its dynamic evolution mode. Since the quantum state lives in the Hilbert space, its gauge freedom cannot be fixed via the Schrödinger equation. It is the gauge freedom of the quantum state, and the quantum Zeno effect can be exhibited in different dynamic evolution modes, revealing its geometrical properties. At the same time, this can be thought of as the manipulating resource to control the dynamic evolution of the quantum system, such as the coherence, entanglement, information reading and writing interactions in the quantum computing processes, etc., via the quantum (anti-)Zeno effect.

To characterize the quantum Zeno capacities of a quantum system, we introduce the quantum Zeno factor, which represents the potential of a quantum system reaching quantum Zeno: a large quantum Zeno factor value means that the quantum system can reach the quantum Zeno effect using less measurement times, or the quantum system can easily yield the Zeno effect. The quantum Zeno factor, as an indicative parameter, can be employed to measure the Zeno quality of a quantum system. This provides a practical way of manipulating the quantum system using the quantum (anti-)Zeno effect.

## Data Availability

The data presented in this study are available upon request from the corresponding author. The data are not publicly available due to privacy restrictions.

## Abbreviations

The following abbreviations are used in this manuscript:

QZE	Quantum Zeno Effect
QAZE	Quantum Anti-Zeno Effect
QZD	Quantum Zeno Dynamics
QSL	Quantum Speed Limit

## Appendix A. Details of the Examples

### Appendix A.1. The Three-Level Quantum System

We consider a three-level system to demonstrate the quantum Zeno effect of the quantum system, which is dependent on the nature of the dynamic evolution mode. The system has two nondegenerate excited states $|E_{1}\rangle$, $|E_{2}\rangle$ and the ground state $|E_{0}\rangle$$\left(E_{0}=0\right)$. The initial state of the quantum system can be written as

(A1) $$\begin{array}{c} |\psi \left(0\right)\rangle =c_{0}|E_{0}\rangle +c_{1}|E_{1}\rangle +c_{2}|E_{2}\rangle , \end{array}$$

and the evolution is governed by the time-independent Schrödinger equation $i\hbar \frac{d}{dt}\psi =H\psi$. Correspondingly, the state at time *t* is of the form (under the assumption ℏ=1):

$$\begin{array}{c} |\psi \left(t\right)\rangle =c_{0}|E_{0}\rangle +c_{1}e^{-iE_{1}t}|E_{1}\rangle +c_{2}e^{-iE_{2}t}|E_{2}\rangle . \end{array}$$

We assume that the evolved state at time $\tau_{⊥}$ is orthogonal to the initial state. Thus, the overlap between the initial and final state should be zero, i.e., $\langle \psi \left(0\right)|\psi \left(\tau_{⊥}\right)\rangle =0$. Consequently, the coefficients are determined by [71]

(A2) $$\begin{array}{cc} p_{1}\sin x_{1}+p_{2}\sin x_{2} & =0\\ p_{0}+p_{1}\cos x_{1}+p_{2}\cos x_{2} & =0. \end{array}$$

where $|c_{j}{|}^{2}=p_{j};\sum_{j}p_{j}=1$, and $x_{j}=E_{j}\tau_{⊥}$ with the indexes j={0,1,2}. The energy uncertainty ΔE and the mean energy 〈E〉 are

(A3) $$\begin{array}{cc} \Delta E & =\sqrt{\langle \psi \left(t\right)|H^{2}|\psi \left(t\right)\rangle -{\left(\langle \psi \left(t\right)|H|\psi \left(t\right)\rangle \right)}^{2}},\\ \; & =\sqrt{p_{1}E_{1}^{2}+p_{2}E_{2}^{2}-{\left(p_{1}E_{1}+p_{2}E_{2}\right)}^{2}},\\ \langle E\rangle & =\langle \psi \left(t\right)|H|\psi \left(t\right)\rangle =p_{1}E_{1}+p_{2}E_{2}, \end{array}$$

(1) The MT dynamic evolution mode

Following the orthogonal condition Equation (A2), we can obtain

(A4) $$\begin{array}{cc} p_{0} & =\frac{\delta }{2},\\ p_{1} & =\frac{1}{2}-\frac{\delta }{4}\left(1+\cos x_{1}\right),\\ p_{2} & =\frac{1}{2}-\frac{\delta }{4}\left(1-\cos x_{1}\right), \end{array}$$

where $p_{0}$ is small, and $x_{2}=\pi +x_{1}-\delta \sin x_{1}$. By employing Equations (A3) and (A4), we can obtain the quantum speed limit of a different evolution mode.

(A5) $$\begin{array}{cc} v_{QSL}^{MT} & =\frac{1}{2}\sqrt{{\left(E_{1}-E_{2}\right)}^{2}+2E_{1}E_{2}\delta },\\ v_{QSL}^{ML} & =\frac{1}{4}\left[\left(E_{1}+E_{2}\right)\left(2-\delta \right)+\left(E_{2}-E_{1}\right)\delta \cos x_{1}\right]. \end{array}$$

Correspondingly, the quantum Zeno factor can be readily written as

(A6) $$\begin{array}{cc} Z_{f}^{ML} & =\frac{4}{\left(E_{1}+E_{2}\right)\left(2-\delta \right)+\left(E_{2}-E_{1}\right)\delta \cos x_{1}},\\ Z_{f}^{MT} & =\frac{2}{\sqrt{{\left(E_{1}-E_{2}\right)}^{2}+2E_{1}E_{2}\delta }}. \end{array}$$

(2) The ML dynamic evolution mode

We consider the three-level quantum system under the assumption that the quantum Zeno factor of the ML evolution mode is larger than the MT evolution mode, $Z_{f}^{ML}>Z_{f}^{MT}$. In this situation, the initial state is the same as Equation (A1); nevertheless, the excited states are $E_{2}=\left(2k+1\right)E_{1}$. Considering the presupposed orthogonal time condition Equation (A2), the population can be expressed as

(A7) $$\begin{array}{cc} \; & p_{0}=\frac{1}{2};\\ \; & p_{1}=\frac{1}{2}\left(1-\frac{\beta }{k^{2}}\right);\\ \; & p_{2k+1}=\frac{\beta }{2k^{2}}, \end{array}$$

where *k* is a positive integer, and β is small.

The quantum speed limit becomes

(A8) $$\begin{array}{cc} v_{QSL}^{MT} & =\sqrt{\frac{E_{1}}{4}+\frac{E_{1}^{2}\beta }{k}-\frac{E_{1}^{2}\beta^{2}}{k^{2}}+2E_{1}^{2}\beta },\\ v_{QSL}^{ML} & =E_{1}\left(\frac{1}{2}+\frac{\beta }{k}\right). \end{array}$$

We can obtain the quantum Zeno factor of MT and ML evolution modes as follows:

(A9) $$\begin{array}{cc} Z_{f}^{ML} & =\frac{2k}{\left(k+2\beta \right)E_{1}},\\ Z_{f}^{MT} & =\frac{2\sqrt{k}}{E_{1}\sqrt{k+4\beta +8k\beta }}. \end{array}$$

### Appendix A.2. Coupled-Qubit System

The Hamiltonian of a coupled-qubit system is given as follows [77,78,79]:

(A10) $$H=-\frac{1}{2}\left(\begin{array}{cccc} \epsilon -s+\kappa & \eta & 0 & -\Delta \eta \\ \eta & -\kappa & \eta & \Delta \epsilon -\Delta s\\ 0 & \eta & \kappa +s-\epsilon & \Delta \eta \\ -\Delta \eta & \Delta \epsilon -\Delta s & \Delta \eta & -\kappa \end{array}\right),$$

with $\epsilon =\epsilon_{1}+\epsilon_{2}$, $\Delta \epsilon =\epsilon_{1}-\epsilon_{2}$, $\eta =\left(\gamma_{1}+\gamma_{2}\right)/\sqrt{2}$, and $\Delta \eta =\left(\gamma_{1}-\gamma_{2}\right)/\sqrt{2}$, where *ϵ*_*ı*_ (*ı*=1,2) are the energy bias, and γ_*ı*_ are the transmission amplitude through the barrier. κ is the strength of the inter-qubit coupling, and $s=X_{1}+X_{2}$ and $\Delta s=X_{1}-X_{2}$, where X_*ı*_ is the coordinate of the bath of harmonic oscillators. The Hamiltonian of two qubits with equal qubit parameters can be written as follows [79]:

(A11) $$H=-\frac{1}{2}\left(\begin{array}{cccc} \epsilon +\kappa & \eta & 0 & 0\\ \eta & -\kappa & \eta & 0\\ 0 & \eta & \kappa -\epsilon & 0\\ 0 & 0 & 0 & -\kappa \end{array}\right),$$

We simply apply Cardano’s formula of the general third-order equation to obtain the eigenvalues and eigenvectors. After some algebraic operations, the eigenvalues can be given by

(A12) $$\begin{array}{cc} \xi_{1} & =\frac{1}{2}\kappa ,\\ \xi_{2} & =2X^{\frac{1}{3}}\cos\vartheta -\frac{1}{6}\kappa ,\\ \xi_{3} & =X^{\frac{1}{3}}\left(-\cos\vartheta +\sqrt{3}\sin\vartheta \right)-\frac{1}{6}\kappa ,\\ \xi_{4} & =X^{\frac{1}{3}}\left(-\cos\vartheta -\sqrt{3}\sin\vartheta \right)-\frac{1}{6}\kappa , \end{array}$$

and the eigenvectors are of the following form:

(A13) $$\begin{array}{cc} \; & |\xi_{1}\rangle ={\left(0,0,0,1\right)}^{T},\\ \; & |\xi_{2}\rangle =\frac{{\left|\eta \right|}^{2}}{g_{8}}{\left(9,-3\frac{g_{3}}{\eta },\frac{g_{4}}{\eta^{2}},0\right)}^{T},\\ \; & |\xi_{3}\rangle =\frac{{\left|\eta \right|}^{2}}{g_{9}}{\left(-\frac{g_{5}}{\eta^{2}},3\frac{g_{6}}{\eta },9,0\right)}^{T},\\ \; & |\xi_{4}\rangle =\frac{\left|\eta |\right|g_{2}|}{g_{7}}{\left(\frac{3g_{1}}{g_{2}},\frac{g_{1}}{\eta },3,0\right)}^{T}, \end{array}$$

where the abbreviations in the eigenvalues and eigenvectors are as follows:

$$\begin{array}{cc} P & =-\frac{1}{12}\epsilon^{2}-\frac{1}{6}\eta^{2}-\frac{1}{9}\kappa^{2},\\ Q & =\frac{1}{12}\epsilon^{2}\kappa -\frac{1}{12}\eta^{2}\kappa -\frac{1}{27}\kappa^{3},\\ X & =\sqrt{-P^{3}},\\ \vartheta & =\frac{1}{3}\arccos\left(-\frac{Q}{X}\right), \end{array}$$

and

$$\begin{array}{cc} g_{1} & =-2\kappa +3\eta +6X^{\frac{1}{3}}\cos\vartheta +6\sqrt{3}X^{\frac{1}{3}}\sin\vartheta ,\\ g_{2} & =-2\kappa -3\eta +6X^{\frac{1}{3}}\cos\vartheta +6\sqrt{3}X^{\frac{1}{3}}\sin\vartheta ,\\ g_{3} & =3\epsilon +2\kappa +12X^{\frac{1}{3}}\cos\vartheta ,\\ g_{4} & =-9\eta^{2}-12\kappa \epsilon -8\kappa^{2}-24\kappa X^{\frac{1}{3}}\cos\vartheta +36X^{\frac{1}{3}}\cos\vartheta +144X^{\frac{2}{3}}\cos^{2}\vartheta ,\\ g_{5} & =8\kappa^{2}-12\kappa \epsilon -12\kappa X^{\frac{1}{3}}\cos\vartheta +12\sqrt{3}\kappa X^{\frac{1}{3}}\sin\vartheta -18\epsilon X^{\frac{1}{3}}\cos\vartheta -36X^{\frac{2}{3}}\\ & -72X^{\frac{2}{3}}\sin^{2}\vartheta +72\sqrt{3}X^{\frac{2}{3}}\cos\vartheta \sin\vartheta +18X^{\frac{1}{3}}\epsilon \sin\vartheta +9\eta^{2},\\ g_{6} & =-2\kappa +3\eta +6X^{\frac{1}{3}}\cos\vartheta -6\sqrt{3}X^{\frac{1}{3}}\sin\vartheta ,\\ g_{7} & =\sqrt{9|g_{1}{|}^{2}{\left|\eta \right|}^{2}+|g_{1}{|}^{2}|g_{2}{|}^{2}+{9|\eta |}^{2}{\left|g_{2}\right|}^{2}},\\ g_{8} & =\sqrt{{81|\eta |}^{4}+9|g_{3}{|}^{2}{\left|\eta \right|}^{2}+{\left|g_{4}\right|}^{2}},\\ g_{9} & =\sqrt{|g_{5}{|}^{2}+9|g_{6}{|}^{2}{\left|\eta \right|}^{2}+81{\left|\eta \right|}^{4}}. \end{array}$$

For this time-independent Hamiltonian, the state vector can be decomposed into the superposition of eigenstates. The initial state can be written as follows:

(A14) $$\begin{array}{c} |\psi \left(0\right)\rangle =c_{1}|\xi_{1}\rangle +c_{2}|\xi_{2}\rangle +c_{3}|\xi_{3}\rangle +c_{4}|\xi_{4}\rangle , \end{array}$$

Correspondingly, the state at time *t* is determined by the unitary operator as follows:

(A15) $$\begin{array}{cc} |\psi (t)\rangle & =\sum_{j=1}^{4}c_{j}e^{-i\xi_{j}t}|\xi_{j}\rangle . \end{array}$$

At this point, the quantum Zeno factor of the ML and MT dynamic evolution modes can be written as follows:

(A16) $$\begin{array}{cc} Z_{f}^{ML} & =\frac{1}{\sum_{j=1}^{4}p_{j}\xi_{j}},\\ Z_{f}^{MT} & =\frac{1}{\sqrt{\sum_{j=1}^{4}p_{j}\xi_{j}^{2}-{\left(\sum_{j=1}^{4}p_{j}\xi_{j}\right)}^{2}}}, \end{array}$$

where $p_{j}={\left|c_{j}\right|}^{2}$.

Considering the SZ mode (for the parallel transporting), the state should obey the parallel condition. The quantum state, after a gauge transformation, can be written as follows:

(A17) $$\begin{array}{c} |\bar{\psi }\left(t\right)\rangle =e^{i\int_{0}^{t}\langle \psi \left(t^{\prime }\right)|H\left(t^{\prime }\right)|\psi \left(t^{\prime }\right)\rangle dt^{\prime }}|\psi \left(t\right)\rangle . \end{array}$$

Equation (A17) keeps the parallel transport rule, $\langle \bar{\psi }\left(t\right)|\dot{\bar{\psi }}\left(t\right)\rangle =0$. By employing the auxiliary state Equation (A17), the geometric can be written as follows:

(A18) $$\varphi_{geo}=\varphi_{total}=\mathrm{Arg}\left\{\langle \psi \left(0\right)|\bar{\psi }\left(t\right)\rangle \right\}.$$

With Equations (A15) and (A17), the geometric phase becomes

(A19) $$\begin{array}{c} \varphi_{geo}=\sum_{j=1}^{4}p_{j}\xi_{j}t+\arctan\frac{Y}{X}. \end{array}$$

where $X=\sum_{j=1}^{4}p_{j}\cos\xi_{j}t$ and $Y=\sum_{j=1}^{4}p_{j}\sin\xi_{j}t$. The changing rate of geometric phase can be expressed as follows:

(A20) $$\begin{array}{c} {\dot{\varphi }}_{geo}=\sum_{j=1}^{4}p_{j}\xi_{j}+\frac{\dot{Y}X-Y\dot{X}}{X^{2}+Y^{2}}. \end{array}$$

The quantum Zeno factor of the SZ mode can be obtained by Equation (A20). The QSL of the parallel transporting state Equation (A17), which is parallel with the geodesic curve connecting the initial and final state, is the changing rate of the geometric phase.
